# Complex S10+S7b Segmentectomy by Pulmonary Ligament Approach Based on B7 Branching Pattern

**DOI:** 10.70352/scrj.cr.25-0562

**Published:** 2025-10-28

**Authors:** Yukitaka Sato, Fumitsugu Kojima, Shinsaku Kabemura, Toru Bando

**Affiliations:** Department of Thoracic Surgery, St. Luke’s International Hospital, Tokyo, Japan

**Keywords:** B7 branching, pulmonary ligament approach, complex segmentectomy, S10 segmentectomy

## Abstract

**INTRODUCTION:**

Based on the results of the JCOG0802 study, segmentectomy is now considered the standard treatment for early-stage lung cancer. However, segmentectomy for right basal segment lesions remains technically challenging because of the complexity of B7 branching. B7 demonstrates 3 distinct branching patterns based on its anatomical relationship with the basal vein (BV): ventral, bilateral, and dorsal. Herein, we report a successful S10+S7b complex segmentectomy for S10 lung cancer with a bilateral B7 branching pattern using a pulmonary ligament (PL) approach.

**CASE PRESENTATION:**

A 70-year-old woman presented with a 15-mm partially solid nodule in the right S10 segment (cT1miN0M0, cStage IA1). 3D-CT revealed bilateral B7 branches crossing the BV. A preoperative bronchoscopic marking with indigo carmine was conducted to guide the intersegmental plane. Three-port video-assisted thoracic surgery was performed via the PL approach. Following V7b dissection, the crossing of B7a and B7b over the BV was clearly identified. The dorsal branch of the B7 (B7b), located ipsilateral and central to the B10, was first divided. Sequential dissections of the veins, bronchi, and arteries at S10 and S7b were then performed. The intersegmental plane was stapled according to the preoperative bronchoscopic markings to ensure adequate margins. The postoperative course was uneventful, and the patient was discharged on POD 6. The pathological examination revealed adenocarcinoma (pT1miN0M0, pStage IA1) with negative margins (20 mm).

**CONCLUSIONS:**

This case may serve as a reference for surgical planning in segmentectomy for right basal lesions according to B7 branching patterns. Even in complex cases with bilateral B7 branching patterns, initial B7b dissection facilitates access to the B10. The PL approach provides not only direct access to the basal segment vasculature and bronchi but also superior visualization of the anatomy of B7 branching.

## Abbreviations

BV
: basal vein
JCOG
: Japan Clinical Oncology Group
PL
: pulmonary ligament

## INTRODUCTION

**Fig. 1:**
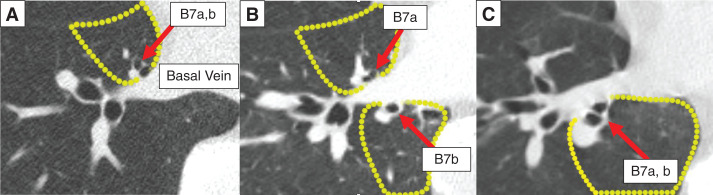
B7 branching is classified as ventral (**A**), bilateral (**B**), or dorsal (**C**), based on its positional relationship with the basal vein (red arrow: B7 branch, yellow dotted line: S7 segment).

Based on the results of the JCOG0802 trial, segmentectomy has become the standard treatment for early-stage lung cancer.^1)^ However, segmentectomy for right basal lesions remains technically challenging because of the complex B7 branching patterns. B7 branching is classified into the ventral, bilateral, and dorsal types, based on its positional relationship with the basal vein (BV)^2)^ (**Fig. 1**). These anatomical variations significantly impact surgical planning for segmentectomy of right basal lesions. Herein, we report a successful case of S10+S7b complex segmentectomy for early-stage S10 lung cancer with bilateral B7 anatomy using a pulmonary ligament (PL) approach.

## CASE PRESENTATION

**Fig. 2:**
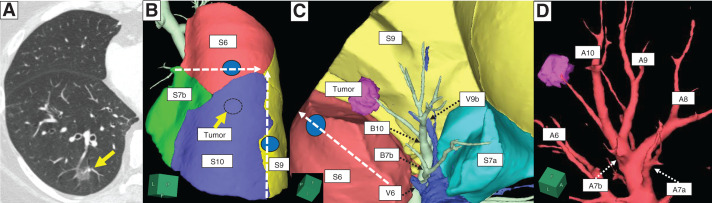
(**A**) CT image showing a 15-mm part-solid nodule in the right S10 segment (yellow arrow: tumor). (**B**) 3D reconstruction CT showed S7b interposed between S6 and S10. Preoperative bronchoscopic marking with indigo carmine was performed for B6c and B9a (2 blue spots) to guide the intersegmental plane (white dotted arrows indicate the estimated intersegmental resection lines). (**C**) 3D-CT imaging from the pulmonary ligament approach showing B7b positioned proximal to B10. Initial dissection of B7b enables easier access to B10. Due to imaging limitations, V6c could not be reliably reconstructed (white dotted arrow: estimated intersegmental resection line; blue spot: bronchoscopic marking for B6c; black dotted arrow: each branch of pulmonary vein and bronchus). (**D**) 3D-CT imaging of pulmonary arteries showing A7b originating from the A9+10, whereas A7a branches from the basal pulmonary artery. B7b, dorsal branch of B7

A 70-year-old woman presented with a 15-mm part-solid nodule (cT1miN0M0, cStage IA1) in the right S10 lung segment detected on follow-up CT after surgery for uterine cancer (**Fig. 2A**). CT identified the B7 bronchus bilaterally, with an incomplete interlobar fissure. Therefore, we chose the PL approach, a method in which the PL is dissected first, followed by sequential division of the pulmonary vein, bronchus, and pulmonary artery. 3D image analysis using Synapse Vincent (Fujifilm Medical, Tokyo, Japan) showed that the S7b was interposed between the S6 and S10 (**Fig. 2B**). To access B10, we first needed to dissect the dorsal branch of B7 (B7b), located ipsilateral and proximal to B10 (**Fig. 2C**). We planned an S10+S7b complex segmentectomy using the PL approach to safely dissect the vasculature and bronchi while ensuring adequate oncological margins. Preoperative bronchoscopic indigo carmine markings were placed at B9a and B6c to guide the intersegmental plane. The resection line was then preoperatively designed using 3D simulation informed by these markings, enabling stapler-based navigational segmentectomy^3)^ (**Fig. 2B**–**2C**).

### Operative course (video)

[Fig F3]

**Fig. 3 F3:**
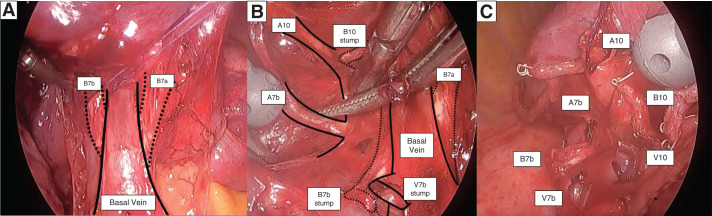
(**A**) After V7b dissection, the crossing of B7a and B7b over the basal vein was clearly visualized using the pulmonary ligament approach. (**B**) After dissection of the veins and bronchi, A7b and A10 were exposed in the deeper field. (**C**) After S10+S7b resection, each stump was clearly identified.

1. Three-port video-assisted thoracoscopic surgery was performed. The ports were placed as follows: a main (utility) port at the 5th intercostal space on the anterior axillary line, an assistant port at the 7th intercostal space on the posterior axillary line, and a camera port at the 8th intercostal space on the midaxillary line. The right lower lung lobe was retracted cranially, and the PL was dissected up to the BV. The V7b was dissected after peripheral exposure of the BV. To improve visualization of deeper structures, the distal stumps of the pulmonary veins and bronchi were ligated with 3-0 Vicryl and used for traction.
2. Crossing of the B7a and B7b over the BV was clearly visualized using the PL approach (**Fig. 3A**). To approach the B10 and effectively perform stapling between the intersegmental planes, an initial dissection of B7b was performed.
3. Dissection was extended to the distal branch of the BV, where V10 was identified and dissected. B10 was identified by bronchoscopy before dissection using a stapler.
4. The A7b originated from the A9+10, whereas A7a branched from the basal pulmonary artery (**Fig. 2D**). The remaining A7b and A10 were dissected (**Fig. 3B**).
5. The intersegmental planes were defined according to the preoperative bronchoscopic markings and stapled along the S9/10 and S6/S7b+S10 boundaries. After resection, the stumps of segments S10 and S7b were clearly identified (**Fig. 3C**).
  The postoperative course was uneventful, and the patient was discharged on POD.
6. The pathological examination revealed adenocarcinoma (pT1miN0M0, pStage IA1) with negative surgical margins (20 mm).

## DISCUSSION

Precise identification of bronchial anatomy is essential to ensure successful segmentectomy. Particularly in B7 branching, approximately 30% of cases exhibit the bilateral B7 branching type.^2)^ This anatomical complexity makes segmentectomy for the basal lesions more challenging. Several reports have described B7 segmentectomy based on branching patterns.^4–6)^ However, approaches to basal segmental lesions based on B7 anatomy remain insufficiently discussed in the current literature.

This case may serve as a reference for surgical planning in segmentectomy for right basal lesions based on B7 branching patterns. For basal lesions, initial dissection of the ipsilateral B7 branch to the lesion enables a safe and technically feasible segmentectomy. In the present case, resection including the dorsal S7 region ipsilateral to the S10 tumor facilitated precise, minimally invasive surgery using the PL approach. The preoperative assessment of B7 branching patterns is crucial for surgical planning.

The PL approach is suitable for basal segment lesions, particularly in cases with incomplete interlobar fissures. Right S10 segmentectomy can also be performed using the interlobar or posterior approach; however, particularly in cases with bilateral B7 branching patterns, the PL approach provides better visualization of the anatomy of the BV, B7a, and B7b. Additionally, this approach allows direct access to the target basal vasculature and bronchi, potentially reducing postoperative air leakage and avoiding unnecessary interlobar manipulation.^7)^ Redo surgeries, such as completion lobectomy, are often challenging because of adhesions caused by the initial surgery.^8)^ This caudal, unidirectional approach could minimize the risk of future interlobar adhesions, which would be advantageous for possible future surgeries.

## CONCLUSIONS

This case may serve as a reference for surgical planning in segmentectomy for right basal lesions based on B7 branching patterns. Even in complex cases with bilateral B7 branching patterns, initial B7b dissection enables effective access to the B10. The PL approach provides not only direct access to the basal segment vasculature and bronchi but also superior visualization of the B7 branching anatomy.

## SUPPLEMENTARY MATERIALS

Supplementary Video Three-port video-assisted thoracoscopic surgery was conducted using the pulmonary ligament (PL) approach. The right lower lobe was retracted cranially, and the PL was dissected up to the basal vein (BV). After V7b dissection, the crossing of the B7a and B7b over the BV was clearly visualized using the PL approach. To approach the B10, an initial B7b dissection was performed. The remaining A7b and A10 cells were subsequently dissected. The intersegmental planes were defined using bronchoscopic markings and stapled along the S9/10 and S6/S7b–S10 boundaries. After resection, the stumps of the pulmonary artery, pulmonary vein, and bronchi of segments S10 and S7b were identified.

## DECLARATIONS

### Availability of data and materials

The data underlying this article will be shared upon reasonable request from the corresponding author.
